# Auto-immune thyroid dysfunction induced by tyrosine kinase inhibitors in a patient with recurrent chordoma

**DOI:** 10.1186/s12885-016-2705-3

**Published:** 2016-08-24

**Authors:** Juliette Eroukhmanoff, Frederic Castinetti, Nicolas Penel, Sebastien Salas

**Affiliations:** 1 Department of Medicine, Division of endocrinology, APHM, Conception Hospital, Marseille, France; 2 Department of Medicine, Division of adult oncology, Oscar Lambret Institute, Lille, France; 3 Aix Marseille Univ, INSERM, U911, Marseille, France; 4 Department of Medicine, Division of adult oncology, APHM, Timone Hospital, Marseille, France

**Keywords:** Auto-immune thyroid dysfunction, Tyrosine kinase inhibitors, Chordoma

## Abstract

**Background:**

While hypothyroidism has frequently been reported with the use of TKIs, the thyroid-stimulating hormone (TSH) suppressing effect of TKIs is rare, except for thyroiditis. We describe a case with progressive recurrent chordoma who initially became hyperthyroid in a context of autoimmunity under sorafenib treatment and later under imatinib treatment.

**Case presentation:**

A 57-year-old man with lumbar chordoma began daily treatment of 800 mg sorafenib. He did not have any other medication or recent iodinated-contrast exposure and his family history was negative for thyroid and autoimmune disease. There was no history of neck pain, irradiation or trauma, recent fever or viral illness. Pre-treatment TSH was normal. After 18 weeks of treatment, the patient presented hyperthyroidism with positive anti-TSH receptor antibodies. More surprisingly, Graves’ disease recurred during treatment with imatinib.

**Conclusion:**

The fact that Graves’ disease occurred after two different TKIs suggests that it could be a rare but important class effect. Anti-TSH receptor antibodies should be systematically measured when TSH decreases in order to avoid the erroneous diagnosis of transient hyperthyroidism due to thyroiditis.

## Background

Tyrosine kinase inhibitors (TKIs) are molecular targeted agents used in the treatment of various cancers. Treatment with TKIs has been associated with changes in thyroid hormone status. While hypothyroidism has frequently been reported with the use of TKIs, hyperthyroidism is less frequent, except with thyroiditis [1–3]. Here, we describe a case with progressive recurrent chordoma who initially became thyrotoxic in a context of autoimmunity under sorafenib treatment and later under imatinib treatment.

## Case presentation

A 57-year-old man with lumbar chordoma was treated initially by two surgical interventions, radiotherapy and proton therapy. In 2011, he began a daily treatment of 800 mg sorafenib in a phase 2 Angionext clinical trial. This study is registered in the European Clinical Trials Register (EudraCT N° 2007-004651-10) and in the ClinicalTrial.gov site (Number: NCT 00874874). He did not have any other medication or recent iodinated-contrast exposure and his family history was negative for thyroid and autoimmune disease. There was no history of neck pain, irradiation or trauma, recent fever or viral illness. Pre-treatment TSH was normal.

After 18 weeks of treatment, his general condition worsened with anorexia, abdominal pain and nausea, leading to a weight loss of 30 % compared to baseline (Grade 4 undernutrition). His test results were suggestive of overt hyperthyroidism [TSH < 0.005 IU/ml (0.27–4.20); free thyroxine (fT4): 34 ng/l (9.32–17.1) and free triiodothyronine (fT3): 10.1 ng/l (2.54–4.36)]. High titers of anti-TSH receptor antibodies were present [31.8 IU/l (N < 1 IU/l)] (Roche Diagnostics, Elecsys 2010, limit of quantification 0.9 U/L). Neck ultrasound showed a voluminous homogeneous gland moderately hypervascularized and without any nodule, and Tc-99 thyroid scintigraphy showed homogeneously increased absorption. These results were highly suggestive of Graves’ disease so the patient was treated with 40 mg carbimazole daily. At this time, sorafenib was discontinued. One month after starting carbimazole, thyroid hormone levels were suggestive of mild hypothyroidism (fT4, 5.7 ng/l; TSH, 6.3 IU/ml). Anti-TSH receptor antibodies were still positive (37.2 IU/l). Carbimazole was maintained at the same dose and L-thyroxin was initiated. Euthyroidism (normal TSH and T4 levels) was obtained one month later, while anti-TSH receptor antibodies were still present though largely decreased (9 IU/L).

The patient was then switched to imatinib 400 mg daily. One month after starting it and while still on fixed doses of carbimazole and L-thyroxin, the patient developed subclinical hyperthyroidism (TSH: 0.14 IU/ml, fT4: 14.9 ng/l, fT3: 6.7 ng/L). Surprisingly, anti-TSH receptor antibodies had risen sharply to 199 UI/l. No extra-thyroid sign of Grave’s disease was observed. Carbimazole was maintained at the same dose while L-thyroxin was rapidly decreased, hyperthyroidism continued to raise (TSH = 0.008 IU/l, fT4 = 30.9 ng/l, T3 = 10.6 ng/l) during the 3 months after beginning imatinib. A mild hypothyroidism at week 106 (TSH 10, fT4 6.6 ng/l, and fT3 3 ng/l) led us to decrease the carbimazole dose and moderately increase the L-thyroxin dose. Anti-TSH receptor antibodies decreased to 9.9 IU/l. Six months later, the patient was euthyroid with the same dose of carbimazole and L-thyroxin, while still on the same dose of imatinib. At the last follow-up 4 months later, anti-TSH receptor antibodies had increased to 37 IU/l and the L-thyroxin dose had to be decreased owing to the recurrence of hyperthyroidism (TSH 0.1 UI/L; fT4 24 ng/l; fT3 10.6 ng/l). Thereafter, the patient presented a tumor progression and imatinib was discontinued. He was euthyroid again one month later, and carbimazole and L-thyroxin doses were progressively decreased.

## Conclusion

TKI-induced hyperthyroidism has been mainly described as a preliminary step before hypothyroidism in some patients with a likely mechanism of destructive thyroiditis. In this setting, hyperthyroidism is always transient and does not necessarily require any treatment. [1, 2, 4]. Miyake et al. reported in a prospective observational trial that 23.9 % of patients with metastatic Renal Cell Carcinoma receiving sorafenib had decreased TSH levels before developing hypothyroidism, which suggested a drug-induced destructive thyroiditis causing thyroid hormone release [5]. The same evolution from hyper- to hypothyroidism was also reported in patients with hepatocarcinoma treated by sorafenib [2].

We report here a case of hyperthyroidism with positive anti-TSH receptor antibodies after treatment with two different successive TKI, sorafenib and imatinib. In our case, hyperthyroidism was due to Graves’ disease as proven by highly positive anti-TSH receptor antibodies, neck ultrasound and scintigraphy. The classical chronology of Graves’ disease is that positive anti-TSH receptor antibodies are detected shortly before the clinical signs. Our patient developed overt hyperthyroidism 18 weeks after starting a TKI. It is highly likely that these antibodies appeared because of the drug, and that the patient first presented sorafenib-induced Graves’ disease. Hyperthyroidism with positive anti-TSH receptor antibodies was also reported in a Turkish patient with a metastatic renal cell cancer treated by sorafenib [6].

More surprisingly, Graves’ disease appeared during treatment with imatinib at a time when the patient was still being treated by anti-thyroid drugs. The major increase in anti-TSH receptor antibodies (from 32 to 199 UI/l) suggests that this occurrence was not due to poor compliance. To our knowledge, this is the first description of such an event occurring with imatinib, which is known to induce hypothyroidism [7–9]. One hypothesis to explain the discrepancy between the large number of patients treated by imatinib and the few cases of Graves’ disease reported until now could be that imatinib is able to induce Graves’ disease only in patients with anti-TSH receptor antibodies, which was the case for our patient who still had positive antibodies when imatinib was initiated.

From an endocrinological viewpoint, the dose needed to control Graves’ disease is also surprising. While the treatment of TKI-induced hypothyroidism can sometimes be challenging with the need for a rapid increase in the dose of L-thyroxin, the treatment of Graves’ disease in our patient seemed rather easy despite the fact that the anti-TSH receptor antibodies were particularly increased. A standard dose of carbimazole finally afforded rapid control of the hyperthyroidism (at its 1st and 2nd occurrences) that was still effective at the most recent follow-up. There is no clear explanation for the final increase in anti-TSH receptor antibodies despite unchanged doses of carbimazole, L-thyroxin and imatinib. One might argue that this easy response could be due to the fact that our patient was actually having thyroiditis rather than Grave’s disease: However, the method of TRAb measurement was highly specific, the scintigraphy showed increased trapping of the radionuclide, and the ultrasound revealed a mildly hypervascular goiter. T3 levels were not as high as expected during the first episode (but this might be due to the altered general condition of the patient), and this could be an evidence for thyroiditis; T3 levels were more consistant with Grave’s disease during the recurrence of thyrotoxicosis. In both cases, TRAb were highly positive, and they were measured in the same department of biology. All these evidence make it more likely the diagnosis of Grave’s disease than thyroiditis. From an oncological viewpoint, the fact that Graves’ disease occurred after two different TKIs suggests that it could be a rare but important class effect. As several studies have also demonstrated impaired thyroid function with sunitinib [10–14], axitinib [15, 16] and motesanib [17], our case report emphasizes the need for thyroid follow-up during any treatment with a TKI in order to screen for both hypothyroidism and hyperthyroidism. Systematically measuring anti-TSH receptor antibodies before TKI initiation is not realistic due to the low frequency of hyperthyroidism, unlike with hypothyroidism. However, anti-TSH receptor antibodies should be systematically assessed whenever TSH levels decrease in order to avoid the erroneous diagnosis of transient hyperthyroidism due to thyroiditis.

Even though hyperthyroidism is rare, diagnosing Graves’ disease is of major importance as it requires prolonged treatment, unlike thyroiditis. Further studies on the mechanism of how TKIs lead to thyroid dysfunction and autoimmunity are needed in order to understand and predict the potential risks for patients on this therapy. There is evidence that various TKIs are able to modulate immune responses. In particular, TKIs might mediate both beneficial and harmful effects on immune cells, and trigger global auto-immunity system, by releasing new tumoral antigenes [18–20]. Moreover, a recent prospective observational cohort study of 27 patients showed novel appearance of antithyroid antibodies in one third of patients under Sunitinib, concluding that TKIs could be able to trigger/exacerbate thyroid autoimmunity [21]. Oncologists and endocrinologists should collaborate closely in the management of patients whenever endocrine side effects of a more and more frequently given treatment, namely TKI, become apparent.

## Abbreviations

fT3: Free triiodothyronine
fT4: Free thyroxine
TKI: Tyrosine kinase inhibitor
TRAb: Anti-thyroid-stimulating hormone antibodies
TSH: Thyroid-stimulating hormone
